# H7N9 influenza A virus activation of necroptosis in human monocytes links innate and adaptive immune responses

**DOI:** 10.1038/s41419-019-1684-0

**Published:** 2019-06-05

**Authors:** Andrew C. Y. Lee, Anna J. X. Zhang, Hin Chu, Can Li, Houshun Zhu, Winger W. N. Mak, Yanxia Chen, Kin-Hang Kok, Kelvin K. W. To, Kwok-Yung Yuen

**Affiliations:** 1 Department of Microbiology, The University of Hong Kong, Hong Kong, China; 2 State Key Laboratory of Emerging Infectious Diseases, Hong Kong, China; 3 Carol Yu Centre for infection, Hong Kong, China; 4 Research Centre of Infection and Immunology, The University of Hong Kong, Hong Kong, China

**Keywords:** Cell death, Influenza virus, Immunopathogenesis, Acute inflammation

## Abstract

We previously demonstrated that avian influenza A H7N9 virus preferentially infected CD14^+^ monocyte in human peripheral blood mononuclear cells (PBMCs), which led to apoptosis. To better understand H7N9 pathogenesis in relation to monocyte cell death, we showed here that extensive phosphorylation of mixed lineage kinase domain-like (MLKL) protein occurred concurrently with the activation of caspases-8, -9 and -3 in H7N9-infected monocytes at 6 h post infection (hpi), indicating that apoptosis and necroptosis pathways were simultaneously activated. The apoptotic morphology was readily observed in H7N9-infected monocytes with transmission electron microscopy (TEM), while the pan-caspase inhibitor, IDN6556 (IDN), accelerated cell death through necroptosis as evidenced by the increased level of pMLKL accompanied with cell swelling and plasma membrane rupture. Most importantly, H7N9-induced cell death could only be stopped by the combined treatment of IDN and necrosulfonamide (NSA), a pMLKL membrane translocation inhibitor, but not by individual inhibition of caspase or RIPK3. Our data further showed that activation of apoptosis and necroptosis pathways in monocytes differentially contributed to the immune response of monocytes upon H7N9 infection. Specifically, caspase inhibition significantly enhanced, while RIPK3 inhibition reduced the early expression of type I interferons and cytokine/chemokines in H7N9-infected monocytes. Moreover, culture supernatants from IDN-treated H7N9-infected monocyte promoted the expression of co-stimulatory molecule CD80, CD83 and CD86 on freshly isolated monocytes and monocyte-derived dendritic cells (MDCs) and enhanced the capacity of MDCs to induce CD3^+^ T-cell proliferation in vitro. In contrast, these immune stimulatory effects were abrogated by using culture supernatants from H7N9-infected monocyte with RIPK3 inhibition. In conclusion, our findings indicated that H7N9 infection activated both apoptosis and necroptosis in monocytes. An intact RIPK3 activity is required for upregulation of innate immune responses, while caspase activation suppresses the immune response.

## Introduction

Influenza A virus infection and replication in host cells induce apoptosis^1^, necroptosis^2^ and pyroptosis^3^. Viral proteins including PB1-F2^4^, NP^5^ and NS1^6^ trigger apoptosis through the intrinsic pathway by mitochondrial-dependent activation of caspase-9^1,7^. At the same time, virus-induced cytokines, for instance Fas/FasL^8^ and tumour necrosis factor-related apoptosis-inducing ligand (TRAIL)^9^ activate extrinsic apoptosis through death receptor/ligand-mediated caspase-8 activation. Influenza virus infection also leads to necroptosis, a form of regulated necrosis. Necroptosis depends on the formation and activation of necrosome, which consists of receptor-interacting protein kinase-1 (RIPK1), -3 (RIPK3), and mixed lineage kinase domain-like (MLKL)^10^. Activation of RIPK1/RIPK3 leads to the phosphorylation of MLKL, which translocates to plasma membrane causing membrane permeabilization and cell death^11^. A recent study showed that influenza H1N1(A/Puerto Rico/8/34, PR8/H1N1) induces necroptosis in murine fibroblast through the detection of viral RNA with the intracellular immune sensor, Z-DNA-binding protein 1 (ZBP1), and subsequent activation of RIPK3^2^. PR8/H1N1 viral protein NP and PB1 have also been reported to interact with ZBP1 to activate RIPK3-dependent necroptosis in fibroblast and bone marrow-derived macrophages^12^.

Apoptosis is a host defense mechanism that can control virus replication. By shrinking apoptotic cells and forming apoptotic bodies, apoptosis restrains inflammatory responses and limits tissue damage^13,14^. In contrast, necroptosis causes cell membrane bursting and releases intracellular contents known as danger-associated molecular patterns (DAMPs) that promote inflammation and trigger the anti-viral immune responses^15^. In this sense, sustainable apoptosis is non-immunogenic, while necroptosis is an immunogenic form of cell death. However, excessive cell death, even in the form of apoptosis, could lead to the destruction of respiratory tissue structure and promote severe inflammatory damages^16,17^, which are associated with increased morbidity and mortality^18,19^. Previous studies indicated that the degree of influenza virus-induced cell death is virus strain and cell type dependent. Specifically, H1N1 and H3N2 viruses activated necroptosis and apoptosis pathways in parallel in fibroblasts and epithelial cells^20^. In addition, seasonal H1N1 viruses induced, while A(H1N1)pdm09 suppressed RIPK3-mediated necroptosis in dendritic cell^21^.

Avian influenza A H7N9 virus is highly pathogenic to human, which causes severe viral pneumonia with case fatality approximately 40%^22,23^. Excessive production of proinflammatory cytokine/chemokine is believed to play key roles in the pathogenesis of H7N9^24,25^. We and others previously demonstrated that the high lethality of H7N9 infection in mice^26^ and in humans case^27,28^ had significantly reduced serum neutralizing antibody titer which indicated a weakened adaptive immune response to H7N9 infection. However, the interplay between H7N9 infection, cell death, and immune response is not fully understood. H7N9 induced apoptosis in human bronchial epithelial cells^29^ and alveolar epithelial cells^30^. Similarly, we previously demonstrated that H7N9 effectively infected human CD14^+^ monocytes and induced massive apoptosis^31^, which might partly explain the weakened adaptive immune responses. However, whether H7N9 virus induces necroptosis in human monocytes and if necroptosis modulates immune responses or pathogenesis in H7N9 infection remain unexplored. Here we report that H7N9 concurrently activates apoptosis and necroptosis pathways that result in massive CD14^+^ monocyte cell death. In addition to cell death, activation of RIPK3 in monocytes upregulates the innate immune responses, while activation of caspase activities restrains these responses.

## Materials and methods

### Virus and reagents

The A/Anhui/1/2013 (H7N9) virus was propagated in 10-day-old specific pathogen-free chicken embryos. Infected allantoic fluid was harvested at 72 h post infection (hpi) and clarified by centrifugation. Viral titer was determined as plaque forming unit (PFU) and 50% tissue culture infectious dose (TCID_50_) on Madin-Darby Canine kidney (MDCK) cells^32^, it was then stored in small aliquots at −80 °C until use. All the experiments involving H7N9 virus were performed in biosafety level 3 laboratory at the department of microbiology, the University of Hong Kong.

The inhibitors and chemicals used were as follow: recombinant human IL4, GM-CSF, TNF-α, z-DEVD-FMK, z-IETD-FMK and z-LEHD-FMK (R&D systems, MN, USA), z-VAD-FMK (InvivoGen, CA, USA), IDN-6556 (HaoyuanChemexpress, Shanghai, China), necrostatin-1 (Abcam, Cambridge, UK), GSK’843 (Aobious, MA, USA), GSK’872 and necrosulfonamide (Millipore, MA, USA) and cycloheximide (Sigma, MO, USA). Neutralizing antibodies to TNF-α (Cell signalling technologies), FASLG (R&D system), TRAIL (Abcam) were used.

### Isolation of CD14^+^ monocytes

Buffy coats blood was obtained from Hong Kong Red Cross Blood Transfusion Service. CD14^+^ monocytes were isolated by positive selection method with human CD14 MicroBeads (Miltenyi Biotec, Germany) as we described previously^31^. Briefly, peripheral blood mononuclear cells (PBMCs) were first isolated by gradient centrifugation in Lymphoprep^TM^ density gradient medium (STEMCELL Technologies, Vancouver, Canada). PBMCs were then incubated with CD14 microbeads for 15 min at 4 °C. The cells were washed once and loaded onto a MACS column placed in the magnetic field. The column was washed three times before the elution of the labelled CD14^+^ monocytes. The purity of the obtained monocytes was confirmed to be more than 95% by flow cytometry (data not shown). The monocytes were cultured in RPMI-1640 complete medium supplemented with GM-CSF (10 ng/ml) and IL4 (10 ng/ml), 10% FBS, 1% penicillin-streptomycin, 1% GlutaMAX, 1 mM sodium pyruvate, 1% non-essential amino acid, and 50 μM 2-mercaptoethanol for the experiments except that FBS was left out during the one-hour viral absorption. Protocol for using buffy coats blood from healthy blood donors was approved by the Institutional Review Board of the University of Hong Kong (ref no. IRB UW16-106).

### H7N9 virus infection of human monocytes

Purified monocytes were inoculated with H7N9 virus at MOI of 2. After one hour incubation at 37 °C, the cells were washed with PBS and further incubated in RPMI-1640 complete medium at 37 °C and 5% CO_2_. For treatment, following inhibitors or chemicals were added to the cells after virus absorption: caspase-3 inhibitor z-DEVD-FMK (20 µM), caspase-8 inhibitor z-IETD-FMK (20 µM), caspase-9 inhibitor z-LEHD-FMK (20 µM), pan-caspase inhibitor IDN-6556 (10 µM), RIPK1 inhibitor necrostatin-1 (30 µM), RIPK3 inhibitors GSK’872 (5 µM) and GSK’843 (5 µM) and pMLKL membrane translocation inhibitor necrosulfonamide (NSA, 5 µM). The cells and culture supernatant with or without treatment were collected at 3, 6, 12, 24 and 48hpi for subsequent analysis. For chemically inducing necroptosis in human monocytes, freshly isolated CD14^+^ monocytes were cultured in complete RPMI1640 medium containing a combination of TNF-α (20 ng/ml), cycloheximide (250 ng/ml), and z-VAD-FMK (20 µM) (TCZ). The cells were collected at 3, 6, 12 and 24 h after treatment for further analysis.

### Cell viability assay

The viability of mock- or H7N9-infected monocytes with or without different inhibitors treatment was determined using CellTiter-Glo® Luminescent cell viability assay (Promega, Wisconsin, US) according to the manufacturer’s protocol. In brief, at pre-determined time points after infection or treatment, the CellTiter-Glo substrate reconstituted with CellTiter-Glo buffer was added directly to the cells in culture plate at an equal volume to culture medium. After incubation at room temperature for 10 min, the luminescence signals were measured on Victor X3 multi-plate reader (PerkinElmer, MA, USA).

### Real-time RT-PCR

H7N9- or mock-infected monocytes were collected at 6, 12 and 24hpi. Total cellular RNA was extracted using a MiniBEST Universal RNA extraction kit (Takara Bio Inc., Shiga, Japan). cDNA was synthesized from 300 ng of total RNA using oligo-dT primer and PrimeScript^TM^ RT kit (Takara). Real-time PCR was performed on a LightCycler 480 real-time PCR system (Roche, Basel, Switzerland) using gene specific primers (listed in Table 1) and SYBR Premix ExTaq (Takara). The expression of GAPDH was also quantified for RNA normalization, the relative expressions of target genes was calculated by ΔΔCt method and expressed as fold changes against untreated mock-infected monocytes.

**Table 1:** Sequences of primers

Gene name	Forward primer (5′ to 3′)	Reverse Primer (5′ to 3′)
*GAPDH*	ATTCCACCCATGGCAAATTC	CGCTCCTGGAAGATGGTGAT
*TNFA*	CAAGGACAGCAGAGGACCAG	TGGCGTCTGAGGGTTGTTTT
*FASLG*	TCCAACTCAAGGTCCATGCC	TTGCAAGATTGACCCCGGAA
*TRAIL*	GCTCGTTAGAAAGACTCCAAGA	CCTCAAGTGCAAGTTGCTCAG
*TNFRSF1A*	TGCAACACTGCCTCACTCTT	GGGTTGAGACTCGGGCATAG
*FAS*	GAGCTCGTCTCTGATCTCGC	CGTAAACCGCTTCCCTCACT
*TRAILR1*	CTGTTGTTGCATCGGCTCAG	GAGACGAAAGTGGACAGCGA
*NOXA*	ATTACCGCTGGCCTACTGTG	ATGTGCTGAGTTGGCACTGA
*PUMA*	CGATTGCGATTGGGTGAGAC	CCTGCTCTGGTTTGGTGAGT
*BAD*	CTTGGGCCCAGAGCATGT	ATGATGGCTGCTGCTGGTT
*BID*	AGGAGCACAGTGCGGATTC	TGCGGAAGCTGTTGTCAGAA
*BAK*	GCAGGCTGATCCCGTCC	CTGCGGAAAACCTCCTCTGT
*BCL2*	CAACATCGCCCTGTGGATGA	GGGCCAAACTGAGCAGAGTC
*RIPK1*	CCTGGAGAGTGCAGAACTGG	CGGCTGTGTCTCAGTCTGTT
*RIPK3*	CAGTGTGCAACAGGCAGAAC	GCATTCCTGGAAGGAGGGTC
*IL6*	GGCTGCAGGACATGACAACT	ATCTGAGGTGCCCATGCTAC
*IL1B*	TTCGAGGCACAAGGCACAA	CCATCATTTCACTGGCGAGC
*IFNA*	AGAATCACTCTCTATCTGAAAGAGAAGAAATA	TCATGATTTCTGCTCTGACAACCT
*IFNB*	AGTAGGCGACACTGTTCGTG	GCCTCCCATTCAATTGCCAC
*MIP1A*	CATTCCGTCACCTGCTCAGAA	GGCTGCTCGTCTCAAAGTAGT
*MIP1B*	GTCTGTGCTGATCCCAGTGA	GCGGAGAGGAGTCCTGAGTA
*RANTES*	AAGGAAGTCAGCATGCCTCT	TAAGCTCCTGTGAGGGGTTG
*GM-CSF*	GGGAGCATGTGAATGCCATC	GGCTCCTGGAGGTCAAACAT
*CD80*	TCCACGTGACCAAGGAAGTG	CTCGTATGTGCCCTCGTCAG
*CD86*	TGGGAATGCTGCTGTGCTTA	TTCAGAGGAGCAGCACCAGA
*BIM*	TGGCCCTTTTGCTACCAGAT	AAGGAGGACTTGGGGTTTGTG
*MCL1*	GAGGAGGACGAGTTGTACCG	GGATCATCACTCGAGACAACGA
*BCL-XL*	AATGTCTCAGAGCAACCGGG	CATCCAAACTGCTGCTGTGC

### Flow cytometry assay

For detection of activated caspase-3 and viral NP protein in monocytes, the cells were collected at 12 and 24hpi and fixed with 4% paraformaldehyde, the cell membranes were permeabilized with 0.1% Triton X-100 for 5 min and then incubated with Alexa Fluor 647-conjugated anti-cleaved caspase-3 antibody (BD Biosciences, CA, USA) or FITC-conjugated anti-influenza A virus NP antibody (Abcam) for 30 min at room temperature. To determine the cell surface expression of differentiation or maturation markers, culture supernatant stimulated cells were collected at 48 or 72 h post-stimulation. The cells were stained with antibodies for 15 min at room temperature and then fixed with 4% paraformaldehyde. Brilliant Violet 421-CD80, APC-CD83, Brilliant Violet 605-CD86, APC-CD3, and corresponding isotype control antibodies (all from Biolegend, San Diego, CA, USA) were used. Stained cells were analysed on LSR Fortessa cell analyser (BD Bioscience), data was analysed using Flowjo software (TreeStar, Inc).

### TUNEL assay

DNA fragmentation in H7N9- and mock-infected monocytes was labelled using Click-iT® Plus Terminal deoxynucleotidyl transferase dUTP nick end labelling (TUNEL) kit (Thermo Fisher Scientific) accordingly to the manufacturer’s protocol. Briefly, the cells were fixed with 4% paraformaldehyde and permeabilized with 0.25% Triton X-100 in PBS. After washed twice with deionized water, the cells were incubated with 50 ul of terminal deoxynucleotidyl transferase reaction mixture for 60 min at 37 °C. The cells were washed again with 3% BSA in PBS and incubated with 50 μl of the Click-iT® Plus TUNEL reaction cocktail for 30 min at 37 °C then mounted with VECTASHIELD medium with DAPI (Vector Laboratories, CA, USA) for microscopy examination.

### Immunofluorescence staining of viral NP protein

As we previously described^31^, the cells were fixed in chilled acetone and methanol (1:1) for 20 min at −20 °C and permeabilized with 0.1% Triton X-100. Mouse anti-influenza NP primary antibody were incubated 37 °C for 1 h and followed by FITC-conjugated donkey anti-mouse IgG secondary antibody (Jackson ImmunoResearch, PA, USA). After washing with PBS the slides were mounted with VECTASHIELD mounting medium with DAPI (Vector Laboratories). The stained cells were examined under fluorescence microscope Nikon80*i* imaging system.

### Western blot

Whole-cell lysates were obtained by lysing the cells in RIPA lysis buffer (50 mM Tris-HCl pH 8, 150 mM NaCl, 1% Triton X-100, 0.5% sodium deoxycholate, 0.1% sodium dodecyl sulphate [SDS]) supplemented with phosphatase inhibitors (5 mM sodium fluoride, 1 mM sodium orthovanadate and 1 mM sodium pyrophosphate) and protease inhibitor cocktail (Thermo Fisher Scientific). The boiled lysates were resolved by sodium dodecyl sulphate-polyacrylamide gel electrophoresis (SDS-PAGE) and transferred onto a nitrocellulose membrane (Thermo Fisher Scientific). The membranes were blocked with 5% non-fat dried milk and incubated with the following specific primary antibody: caspase-3 (Abcam), caspase-8 (Cell signalling technology), caspase-9 (Cell signalling technology), FASLG (Abclonal), TRAIL (Abcam), TNF-α (Cell signalling technology), PUMA (Abcam), MLKL (Millipore), phosphorylated MLKL (Abcam) followed by HRP-conjugated goat anti-mouse, rabbit or rat secondary antibodies (Thermo Fisher Scientific) and WesternBright ECL solution HRP substrate (Advansta, CA, USA). The membranes were stripped and re-probed with anti-β-actin antibody (Sigma) as internal control for protein loading.

### Transmission electron microscopy (TEM)

Infected or mock-infected monocytes were washed and fixed in 2.5% glutaraldehyde at 4 °C overnight. The cells were then detached from culture plate using cell scraper and post-fixed with 1% osmium tetroxide. The resin-embedded samples were processed into ultrathin sections using Ultracut UCT Ultramicrotomy (Leica, Wetzlar, Germany). The samples were stained with uranyl acetate and lead citrate and examined under Philips CM100 transmission electron microscope.

### Monocyte in vitro differentiation

The culture supernatants of H7N9-infected monocytes with or without treatment were collected at 24 hpi and UV inactivated for 10 min with UVP ultraviolet crosslinker CL-1000 (Analytik Jena, CA, USA). The UV-inactivated supernatants were diluted 1:1 with fresh culture medium. Freshly isolated CD14^+^ monocytes were incubated with diluted supernatant for 72 h. Cells were then harvested for flow cytometry determination of cell surface expression of CD80, CD83 and CD86.

### Dendritic cell maturation assay

To generate monocytes-derived dendritic cell (MDCs) in vitro, purified CD14^+^ monocytes were cultured in RPMI1640 complete medium containing recombinant human IL4 (10 ng/ml) and GM-CSF (10 ng/ml) for 6 days, during which culture medium was changed every 2 days. The cells were differentiated into immature DC^33^. UV-inactivated culture supernatant from H7N9-infected monocytes were diluted 1:1 with fresh medium without growth factors. MDCs were incubated with 1 ml of diluted supernatant for 48 h and LPS (100 ng/ml) was used as control for dendritic cell maturation induction^33^. Cells were harvested for flow cytometry assay to determine the expression of CD80, CD83 and CD86.

### Allogeneic T cells proliferation assay

Allogeneic CD3^+^ T cells were isolated with Pan T cell isolation kit (Miltenyi Biotec) and labelled with 2 µM of CellTrace CFSE solution (Thermo Fisher Scientific) in PBS at 37 °C for 15 min. Labelled cells were washed once with pre-warmed RPMI-1640 medium containing 10% FBS and co-cultured with in vitro induced dendritic cells at DC/T cell ratio of 1:3 (1 × 10^5^ DC + 3 × 10^5^ naïve T cell) for 4 days and 6 days. T cell proliferation was determined by measuring the fluorescence of CFSE dye by flow cytometry.

### Statistics

All statistical analyses were computed using Prism 7.0 (GraphPad Software Inc., CA, USA). Statistical analysis between the groups was performed by Student’s t-test or one-way ANOVA. *P* values of <0.05 were considered to be statistically significant.

## Results

### H7N9 infection of human monocytes activated both intrinsic and extrinsic apoptosis pathways

**Fig. 1:**
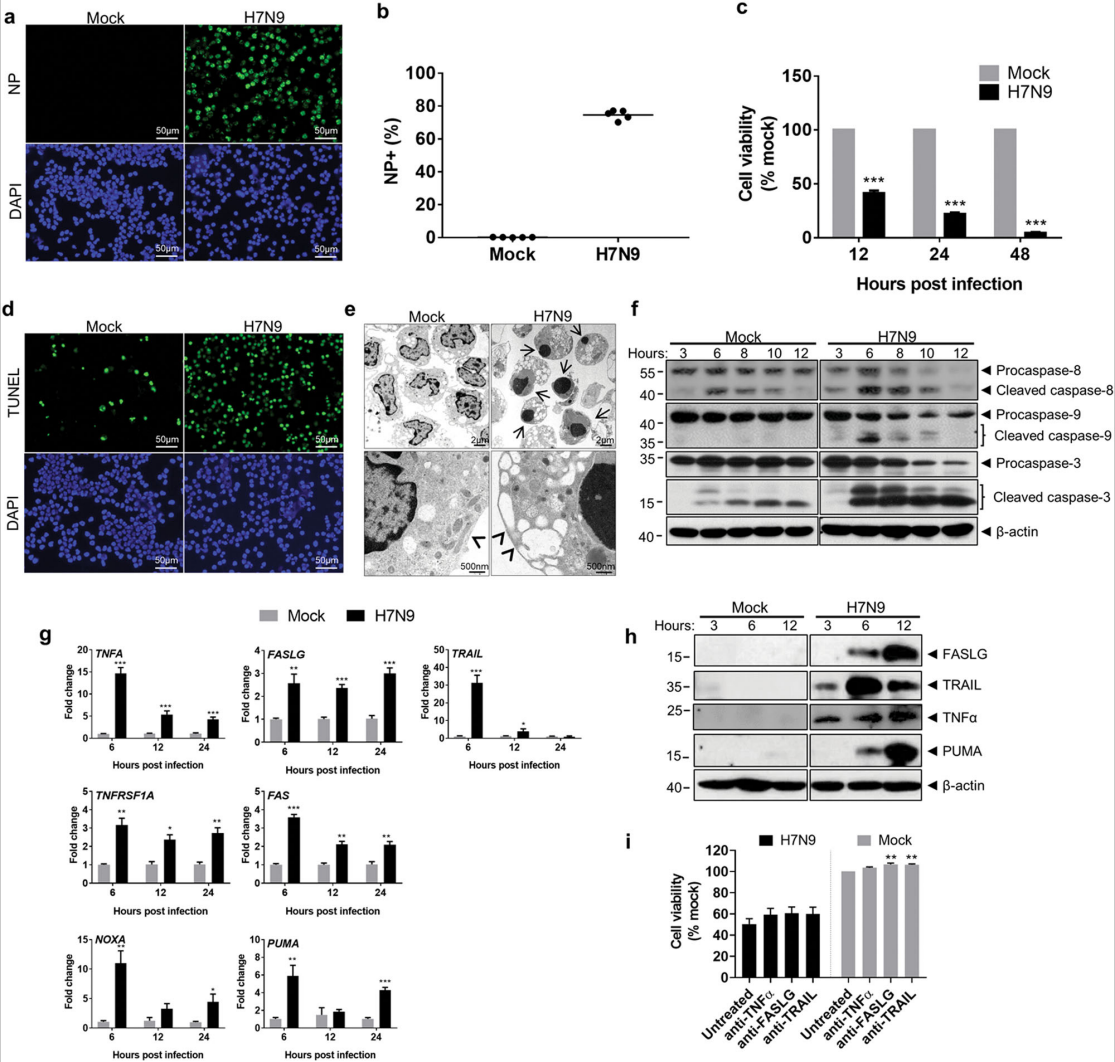
H7N9 infection and induction of apoptosis in human CD14^+^ monocytes. Purified CD14^+^ monocytes were infected with A/Anhui/1/2013 (H7N9) at MOI of 2. **a** Immunofluorescence stained influenza A viral NP protein in H7N9- and mock-infected cells at 12hpi. Representative image of mouse anti-NP protein antibody and FITC-conjugated Donkey anti-mouse IgG stained cells (green) mounted with DAPI (blue). Original magnification of 400x. **b** Percentage of NP positive cells quantified by flow cytometry at 12hpi (*n* = 5 donors). **c** Percentage of viable cells determined at 12, 24 and 48hpi by CellTiter-Glo luminescent cell viability assay. For comparison, viability of mock-infected cells at each time point was taken as 100%. Error bars indicate standard error of the mean. ***p < 0.001 when compared with mock-infected control by student’s *t* test (*n* = 4 donors). **d** Representative images of TUNEL labelled (green) and DAPI (blue) stained H7N9- or mock-infected monocytes at 12hpi. Original magnification 400x. **e** Representative transmission electron microscopy (TEM) images of H7N9- or mock-infected monocytes at 12hpi. The H7N9-infected monocytes were shrunken in size with compacted chromatin (arrows), but intact plasma membrane (arrowheads). Original magnification 1200x and 6000x. **f** Western blot detected cleavages of caspase-8, caspase-9 and caspase-3 in H7N9- or mock-infected monocytes. The expression of β-actin protein was used as the control for equal amount of sample loading. **g** Real-time RT-PCR determined the relative expression of *TNFA*, *FASLG*, *TRAIL*, *TNFRSF1A*, *FAS*, *NOXA* and *PUMA* in H7N9-infected monocytes at 6, 12 and 24hpi. Fold of changes compared to mock-infected monocytes at 6hpi which was taken as 1. Data represented mean of two independent experiments (*n* = 4 donors). Error bars indicate standard error of the mean. *p < 0.05; **p < 0.01; ***p < 0.001 when compared with mock-infected cells by student’s *t* test. **h** Western blot determined the expression of FASLG, TRAIL, TNFα, PUMA protein in H7N9- or mock-infected monocytes at indicated time points. β-actin was used as loading control. **i** Cell viability after neutralizing antibody treatment of H7N9- or mock-infected monocyte determined by CellTiter-Glo luminescent assay. Error bars indicate standard error of the mean. **p < 0.01 when compared with untreated cells by one-way ANOVA (*n* = 4 donors)

Purified CD14^+^ monocytes were inoculated with A/Anhui/1/2013 H7N9 virus at a multiplicity of infection (MOI) of 2 as we previously described^31^. At 12 h post infection (hpi), viral nucleoprotein (NP) was detected by immunofluorescence staining (Fig. 1a), with 77.2% of the cells expressing NP when quantified by flow cytometry (Fig. 1b). The cell viability decreased progressively comparing with mock-infected cells (Fig. 1c). TUNEL staining showed abundant positive cells at 12hpi (Fig. 1d). Under TEM, most cells appeared to have condensed chromatin (Fig. 1e, arrows), cytoplasm shrinkage, and intact plasma membranes (Fig. 1e, arrow heads), compatible with the apoptotic morphology.

Influenza virus infection can trigger both extrinsic and intrinsic apoptosis pathways^34^. Western blot analysis of H7N9-infected monocytes showed reduction of procaspase-8, -9, and -3 starting from 6hpi, while the cleaved forms of caspase-8 (p41/p43), -9 (p35/p37), and -3 (p17/p19) were detected (Fig. 1f). The expressions of death ligands and death receptors involved in the extrinsic pathway including *TNFA*, *FASLG*, *TRAIL*, *TNFRSF1A*, *FAS*, were increased from 6hpi at both mRNA level (Fig. 1g) and protein level (Fig. 1h). The expression of BH3-only proapoptotic proteins, *PUMA* and *NOXA*, involved in the intrinsic pathway were also increased (Fig. 1g, h). The expression of a number of other proteins in the BCL-2 family was also evaluated but was not significantly upregulated at mRNA level (Supplementary Fig. S1). Notably, neutralization of individual stimulants including TNF-α, FASLG and TRAIL by neutralizing antibody did not rescue the viability of H7N9-infected monocytes (Fig. 1i).

### Pan-caspase inhibition altered cell death mode in H7N9-infected monocytes

**Fig. 2:**
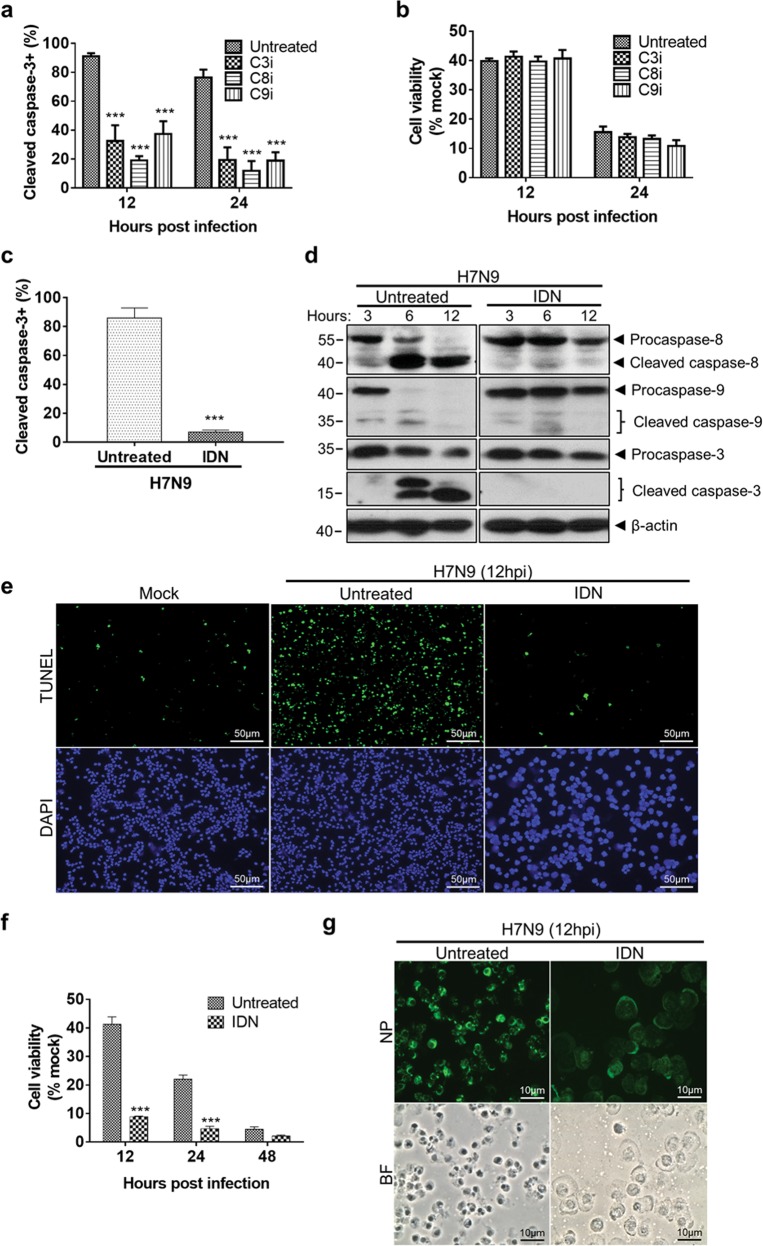
Caspases inhibitors treatment did not protect H7N9-infected monocytes from cell death. Purified CD14^+^ monocytes inoculated with A/Anhui/1/2013 (H7N9) virus at MOI of 2, after 1 h viral absorption at 37 °C, the following caspase inhibitors were added to the culture individually and further incubated: 20 µM of caspase-3 inhibitor (z-DEVD-FMK, C3i), caspase-8 inhibitor (z-IETD-FMK, C8i) or caspase-9 inhibitor (z-LEHD-FMK, C9i) and pan-caspase inhibitor IDN6556 (IDN, 10 µM). **a** Flow cytometry assay determined percentage of cleaved caspase-3 expressing cells in H7N9-infected monocytes treated or untreated with caspase inhibitors at 12 and 24hpi (*n* = 6 donors). **b** Cell viability determined by CellTiter-Glo luminescent assay at 12 and 24hpi (*n* = 6 donors). **c** Percentages of cleaved caspase-3 expressing cells in H7N9-infected monocytes treated or untreated with IDN at 12hpi (*n* = 8 donors). Error bars indicate standard error of the mean. ***p < 0.001 when compared with untreated control by one-way ANOVA. **d** Western blot determined cleavage of caspase-8, -9, -3 in H7N9-infected monocytes treated or untreated with IDN at 3, 6 and 12hpi. β-actin protein was used as the control for sample loading. **e** Representative images of TUNEL labelled (green) and DAPI (blue) stained mock- or H7N9-infected monocytes treated or untreated with IDN at 12hpi. Original magnification of 200x. **f** Cell viability of H7N9-infected with or without IDN treatment at 12, 24 and 48hpi (*n* = 4 donors). Error bars indicate standard error of the mean. ***p < 0.001 when compared with untreated control by one-way ANOVA. **g** Morphology examination of infected monocytes with or without IDN treatment at 12hpi. Representative images of immunofluorescence stained influenza A NP protein (green, upper) and bright field (BF, lower) images of the cells. Original magnification 400x

Next, we evaluated whether H7N9-induced monocyte cell death could be inhibited by chemical inhibitor of caspase-8 (z-IETD-FMK, C8i, 20 µM), caspase-9 (z-LEHD-FMK, C9i, 20 µM), or caspase-3 (z-DEVD-FMK, C3i, 20 µM). Our results showed that all three inhibitors suppressed caspase-3 cleavage at 12 and 24 hpi (Fig. 2a), but failed to improve the viability of the infected monocytes (Fig. 2b). We then evaluated the effect of a pan-caspase inhibitor, IDN6556 (IDN, 10 µM), on H7N9 infected monocyte. Again, caspase-3 cleavage was significantly reduced from 86.1% to 6.9% by IDN treatment at 12hpi (Fig. 2c). By Western blot detection, the amount of cleaved caspase-3, -8 and -9 were largely diminished by IDN treatment at 3, 6 and 12hpi (Fig. 2d). Together with the significant reduction of TUNEL positive cells at 12hpi (Fig. 2e), these data indicated that IDN treatment effectively inhibited apoptosis in H7N9-infected monocytes. However, the viability of H7N9-infected monocytes was significantly reduced after IDN treatment (Fig. 2f). Interestingly, the IDN-treated H7N9-infected monocytes displayed an enlarged and swollen cell morphology, which was different from the typical apoptosis morphology of the untreated H7N9-infected monocytes (Fig. 2g). These results indicated that pan-caspase inhibition promoted a switch of the mode of cell death from mainly apoptosis to necrosis.

### H7N9 infection activated necroptosis pathway concurrently with apoptosis in human monocytes

**Fig. 3:**
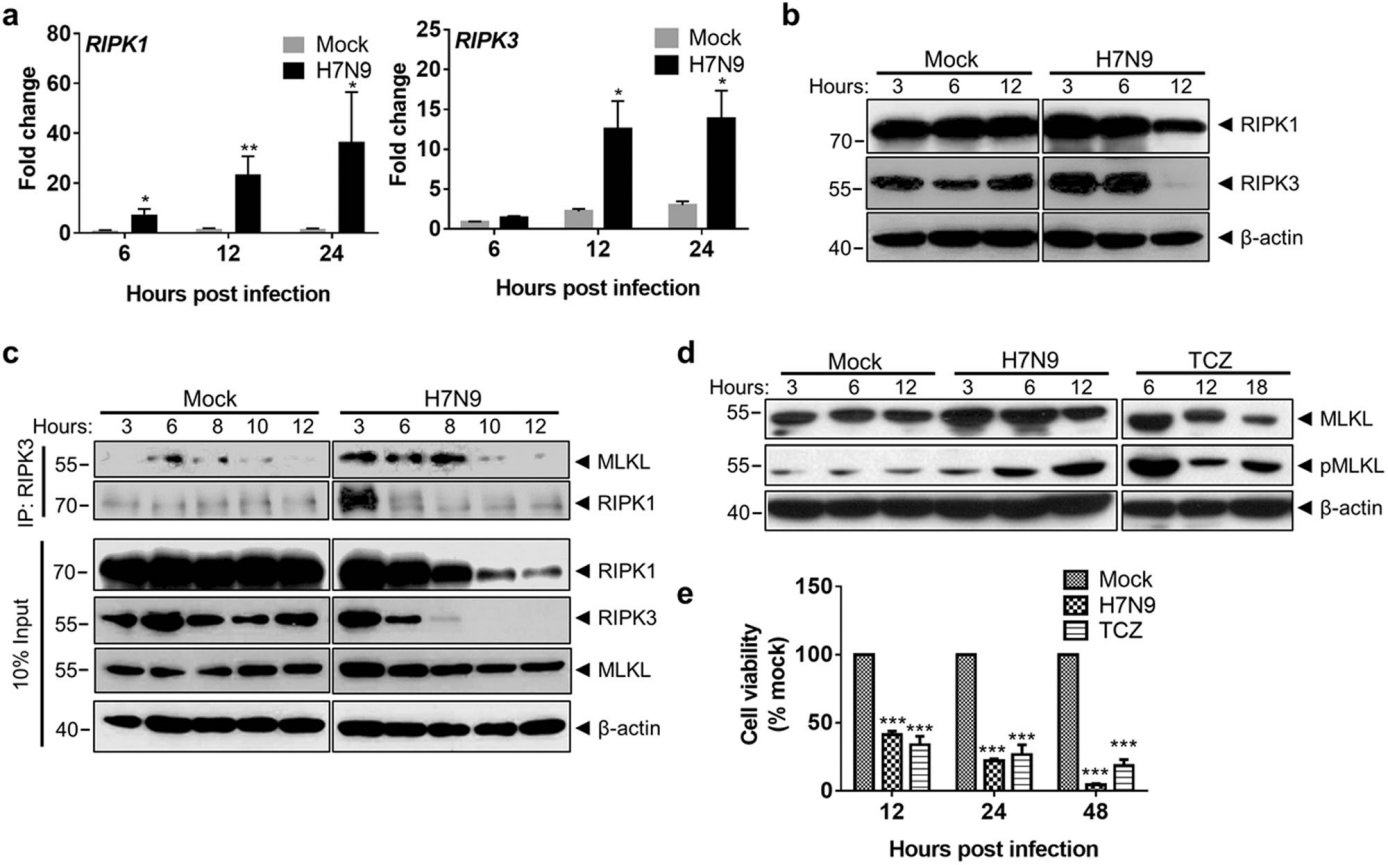
H7N9 infection upregulated RIPK1/RIPK3 expression and MLKL phosphorylation in monocytes. **a** Real-time RT-PCR determined relative expression of *RIPK1* and *RIPK3* in H7N9-infected monocytes. Fold of changes compared with that of mock-infected monocytes at 6hpi which was taken as 1 (*n* = 8 donors). Error bars indicate standard error of the mean. *p < 0.05; **p < 0.01 when compared with mock-infected cells by student’s *t* test. **b** Western blot determined expression of RIPK1 and RIPK3 proteins in H7N9- or mock-infected monocytes at indicated hpi. β-actin was used as sample loading control. **c** Anti-RIPK3 antibody co-immunoprecipitated RIPK1/MLKL from H7N9 infected monocytes at indicated hours post infection. **d** Western blot determined MLKL and pMLKL (S358) in H7N9-infected monocytes or monocytes treated with TCZ combination (TNFα (20 ng/ml), cycloheximide (250 ng/ml) and z-VAD-FMK (20 µM)). β-actin was used as loading control. **e** Viability of H7N9-infected or TCZ-treated monocytes at indicated times. (*n* = 4 donors). Error bars indicate standard error of the mean. ***p < 0.001 when compared with the mock-infected control by one-way ANOVA

**Fig. 4:**
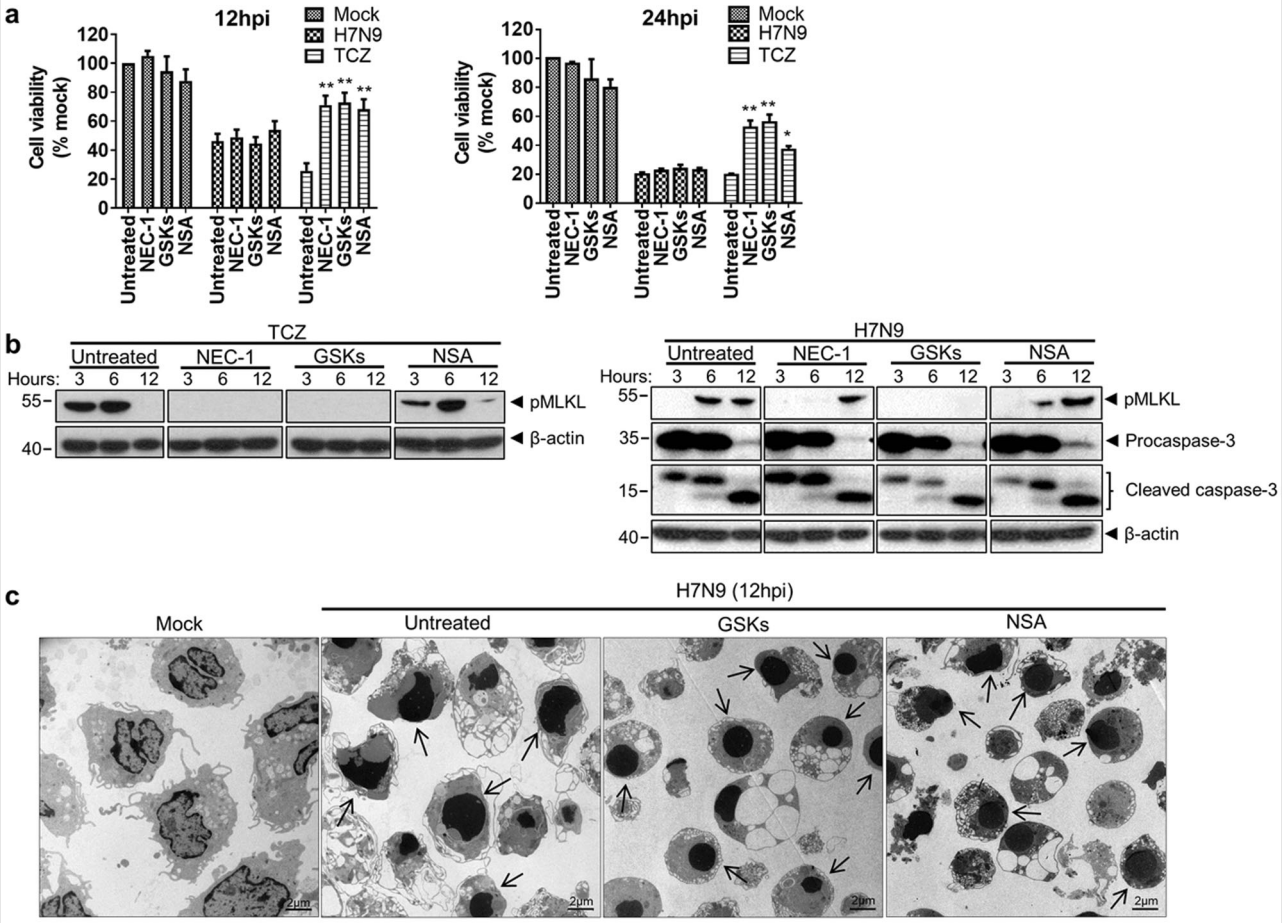
Necroptosis inhibitors did not stop H7N9-infected monocyte death. Necroptosis inhibitors NEC-1 (30 µM), GSKs (GSK’872 5 µM + GSK’843 5 µM), or NSA (5 µM) were added to H7N9-infected cells after 1 h absorption of the virus, and further incubated. The cells were collected at 3, 6, 12 and 24hpi for following analysis. **a** Cell viability of H7N9- or mock-infected cells treated with NEC-1, GSKs or NSA at 12 and 24hpi. Monocytes treated with TCZ was used as a control. Error bars indicate standard error of the mean (*n* = 8 donors). *p < 0.05; **p < 0.01 when compared with untreated monocytes by one-way ANOVA. **b** Western blot determined expression of pMLKL in TCZ-treated monocytes (left); pMLKL and caspase-3 cleavage in H7N9-infected monocytes with or without necroptosis inhibitors treatment (right). **c** Representative TEM images of mock-infected and H7N9-infected cell treated with GSKs or NSA at 12hpi. The untreated H7N9-infected monocytes were shrunken in size, with compacted chromatin (arrows). Same morphologies showed in GSKs- or NSA-treated H7N9-infected monocytes (arrows). Original magnification 1200x

RIPK1 and RIPK3 are two key molecules involved in the activation of the necroptosis pathway^35,36^. We detected quick upregulation of RIPK1 and RIPK3 mRNA in H7N9-infected monocytes from 6hpi onward (Fig. 3a). However, the protein level of RIPK1 was decreased upon infection while the amount of RIPK3 protein was increase at 3hpi and decreased at 12hpi when compared with mock-infected cells (Fig. 3b). Co-immunoprecipitation using RIPK3 as the bait showed an increased amount of RIPK1 and MLKL protein from H7N9-infected monocyte lysate at 3 and 6hpi (Fig. 3c). At the same time, we detected a gradually increased level of phosphorylated mixed lineage kinase domain-like protein (pMLKL), an effector protein inducing necroptosis (Fig. 3d). These results suggested that H7N9 infection activated RIPK1/3 and the necroptosis pathway. As a positive control, TCZ (TNF-α [20 ng/ml], cycloheximide [250 ng/ml], and z-VAD-FMK [20 µM]) treatment significantly upregulated pMLKL (Fig. 3d) and reduced cell viability in monocytes (Fig. 3e), which confirmed that human primary monocytes supported necroptosis in vitro. We next examined the effect of necroptosis inhibitors on H7N9-induced cell death. The results showed that individual treatment with RIPK1 inhibitor necrostatin-1 (NEC-1), RIPK3 inhibitors (GSK’872 + GSK’843; GSKs), or pMLKL membrane translocation inhibitor, necrosulfonamide (NSA), did not improve the viability of H7N9-infected monocytes at 12 and 24hpi despite they significantly inhibited TCZ-induced necroptosis (Fig. 4a). Western blot analysis showed that NEC-1 or GSKs treatment completely diminished pMLKL in TCZ-treated monocytes (Fig. 4b, left). In H7N9-infected monocytes, NEC-1 transiently blocked, while GSKs completely abolished MLKL phosphorylation (Fig. 4b, right), indicating that GSKs treatment effectively inhibited RIPK3 kinase activity in H7N9-infected monocytes. Expectedly, NSA did not alter the protein level of pMLKL^37^. Notably, the level of caspase-3 cleavage in H7N9-infected monocytes was not altered after NEC-1 or GSKs treatment (Fig. 4b, right). In addition, TEM images of infected monocytes predominantly illustrated apoptotic morphologies in the presence of RIPK3 inhibitors (Fig. 4c). These results indicated that RIPK3 kinase activity was not required for H7N9-induced apoptosis, and individual inhibition of necroptosis by RIPK3 kinase inhibitors or pMLKL inhibitor could not prevent H7N9-induced cell death in monocytes.

### Pan-caspase inhibition promoted necroptosis in H7N9-infected monocytes

**Fig. 5:**
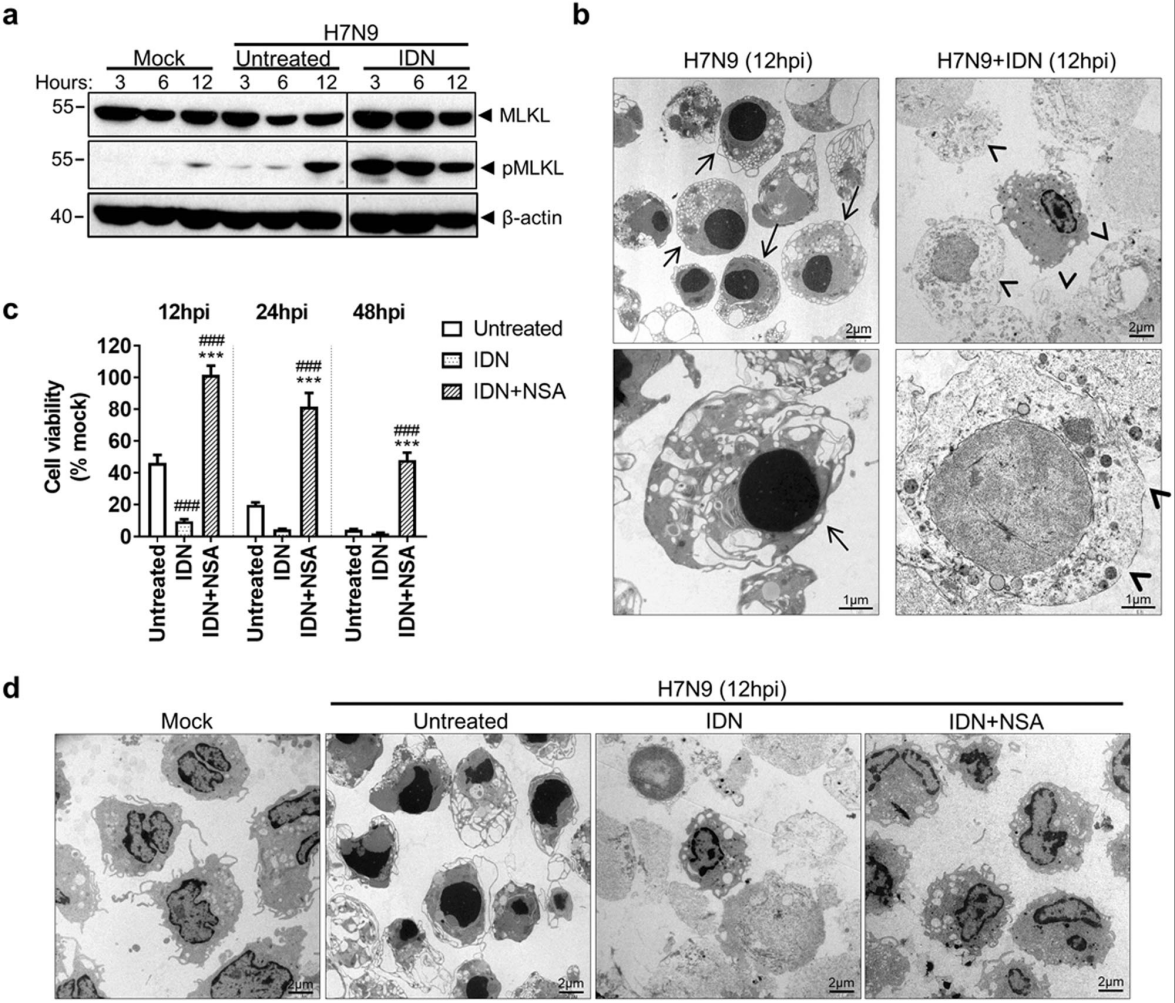
Pan-caspase inhibitor IDN treatment increased pMLKL and switched H7N9-infected monocytes to necroptosis. Monocytes inoculated with A/Anhui/1/2013 (H7N9) virus at MOI of 2, after 1 h viral absorption at 37 °C, IDN or IDN combined with NSA (IDN 10 µM + NSA 5 µM) were added to the culture. The infected cells were further incubated for 3, 6, 12, 24 or 48 h and collected for the following analysis. **a** Western blot determined MLKL and pMLKL after IDN treatment. IDN increased the expression of pMLKL in H7N9-infected monocytes. **b** Representative TEM images of H7N9-infected monocytes with or without IDN treatment at 12hpi. The untreated H7N9-infected monocytes were shrunken in size, with compacted chromatin (arrows), but intact plasma membrane. IDN-treated cells showed cell swelling and rupture of plasma membrane (arrowheads). Original magnification 1200x and 2950x. **c** Cell viability of H7N9-infected monocytes treated with IDN or IDN+NSA determined at 12, 24, 48hpi. (*n* = 4 donors). ^###^*p* < 0.001 when compared with untreated; ***p < 0.01 when compared with the IDN-treated monocytes by one-way ANOVA. **d** Representative TEM images of H7N9-infected monocytes with or without IDN or IDN+NSA treatment at 12 hpi. H7N9-infecetd monocytes treated with IDN showed cell swelling and rapture of plasma membrane. Cells under IDN+NSA treatment showed similar morphologies as mock-infected cells. Original magnification 1200x

IDN treatment accelerated the death of H7N9-infected monocytes, which suggested pan-caspase inhibition modulated the mode of cell death. We found that IDN increased the concentration of pMLKL at 3 and 6hpi (Fig. 5a). Meanwhile, TEM images of IDN-treated H7N9-infected monocytes at 12hpi showed cell swelling and plasma membrane bursting (arrowheads), indicating necrotic cell death (Fig. 5b, right panels). Furthermore, the combined treatment of IDN and NSA protected cell viability to 100%, 81.7% and 48.1% at 12, 24 and 48hpi, respectively (Fig. 5c). This was further supported by TEM, as similar cell morphologies were observed between IDN + NSA-treated H7N9-infected monocytes and mock-infected cells (Fig. 5d). These results indicated that IDN promoted necroptotic cell death and only the combined inhibition of pan-caspase activity and pMLKL membrane translocation could block H7N9-induced cell death in monocytes. In addition, our data suggested that in the presence of IDN treatment, NEC-1 or GSKs could no longer effectively inhibit MLKL phosphorylation and did not improve cell viability. This may be due to the elevated activities of RIPK1 and RIPK3 upon caspase inhibition (data not shown).

### Necroptosis upregulated but apoptosis restrained cytokine/chemokine responses in H7N9-infected monocytes

**Fig. 6:**
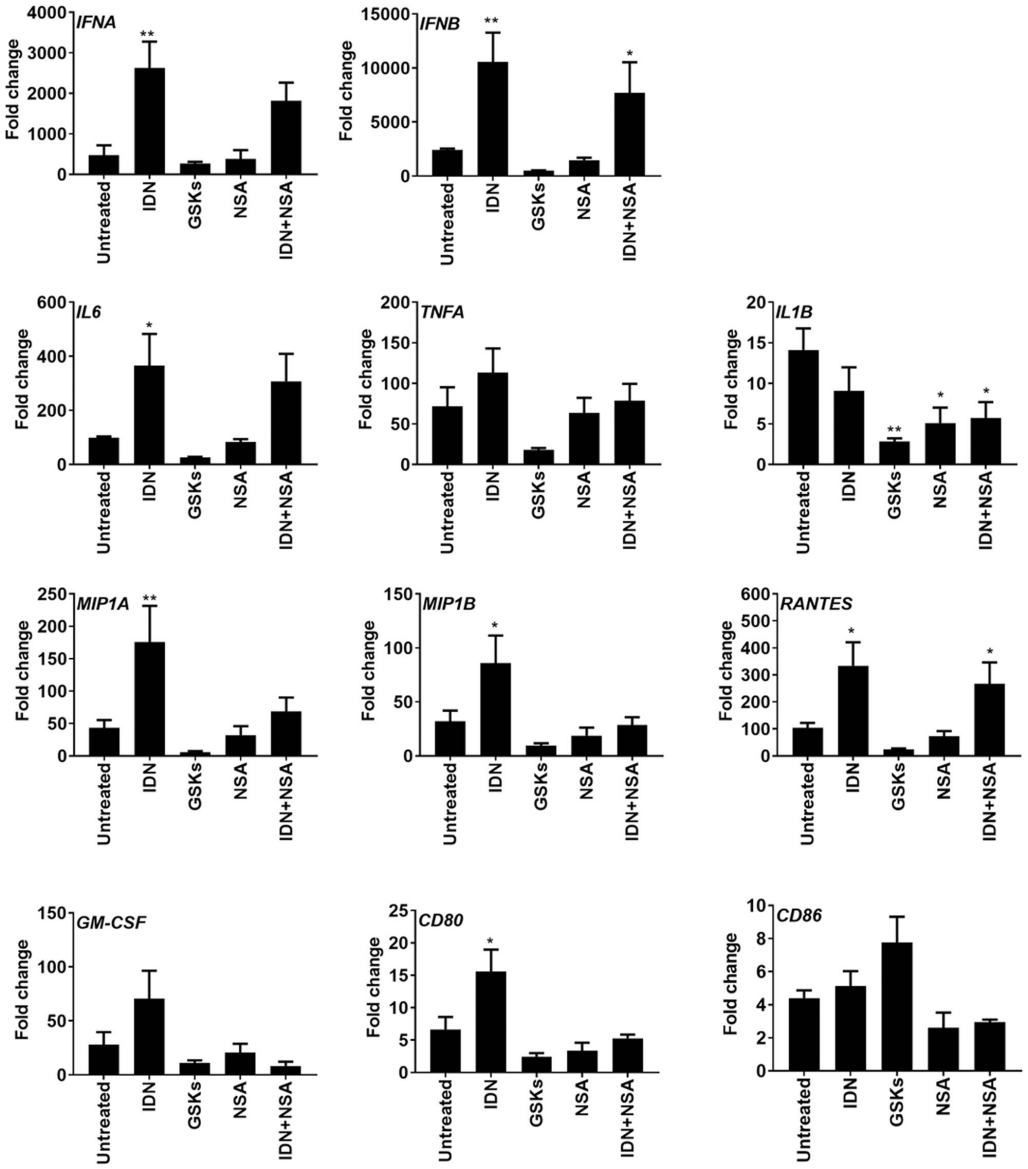
Real-time RT-PCR determined cytokine/chemokine gene expressions in H7N9-infected monocytes with or without treatment. Monocytes inoculated with H7N9 virus (MOI of 2), after virus absorption at 37 °C for one hour, single treatment with IDN (10 µM), GSKs (GSK’872 5 µM + GSK’843 5 µM), NSA (5 µM), or combination treatment with IDN + NSA (IDN 10 µM + NSA 5 µM) were added to the culture for 6 h. Real-time RT-PCR determined relative gene expression for *IFNA/B*, *IL6, TNFA, IL1B*, *MIP1A, MIP1B, RANTES*, as well as *GM-CSF* and co-stimulatory molecules *CD83* and *CD86* were showed as fold of changes comparing with mock-infected untreated control. Data represented mean of two independent experiments (*n* = 4 donors). Error bars indicate standard error of the mean. *p < 0.05; **p < 0.01; ***p < 0.001 when compared with untreated H7N9-infected cells by one-way ANOVA

**Fig. 7:**
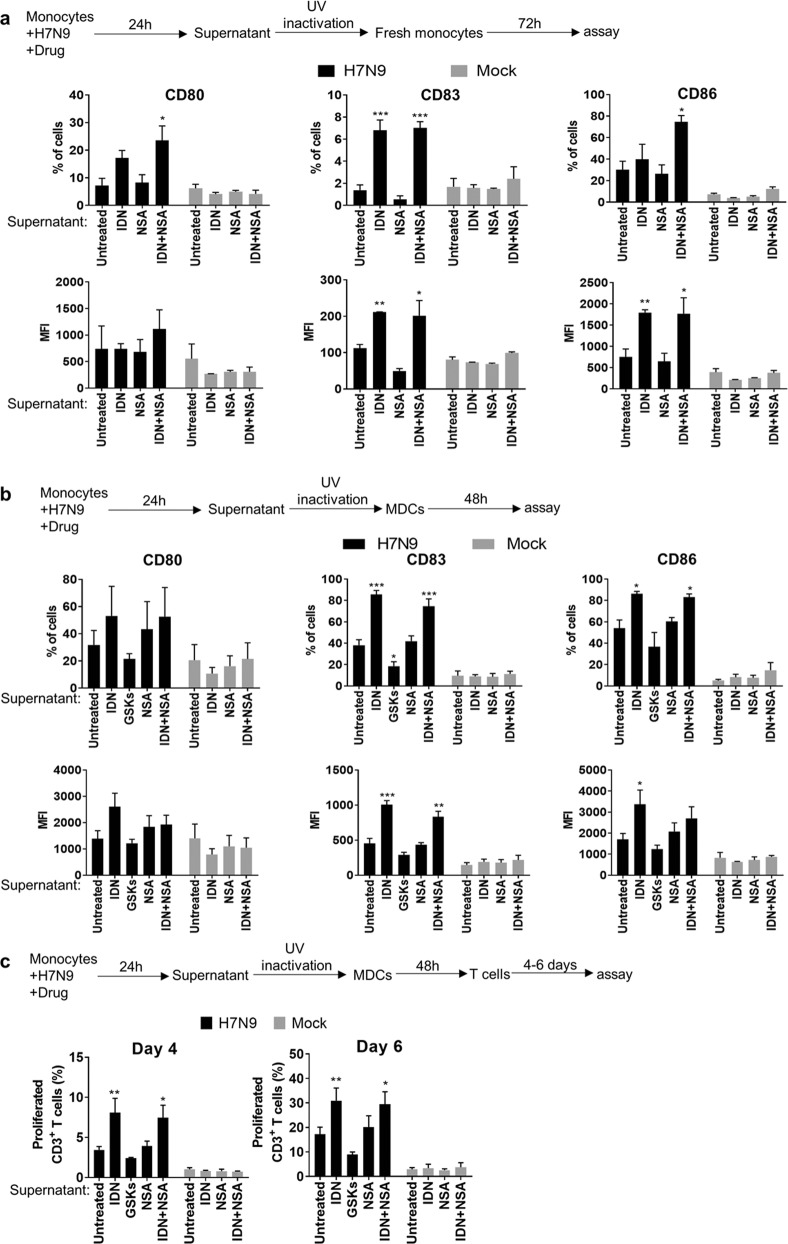
Culture supernatant from H7N9-infected monocytes with IDN treatment stimulated freshly isolated CD14^+^ monocytes or monocytes-derived dendritic cells (MDCs) responses. **a** Upper panel indicated the experimental protocol. Culture supernatant were collected from H7N9- or mock-infected monocytes with or without treatment by IDN, NSA or IDN+NSA at 24 hpi and UV inactivated. The supernatant was diluted with complete culture medium at 1:1 ratio and added to freshly isolated monocytes for further incubation of 72 h. Lower panel showed flow cytometry determined co-stimulator molecule CD80, CD83 and CD86 expressed on monocytes. The percentage of cells expressing surface CD80, CD83 or CD86 and mean fluorescence intensity (MFI) were shown. Data presented are mean of two independent experiments (*n* = 3 donors). Error bars indicate standard error of the mean. *p < 0.05; **p < 0.01; ***p < 0.001 when compared with monocytes stimulated with untreated H7N9-infected monocytes culture supernatant by one-way ANOVA. **b** Upper panel is the protocol for culture supernatant stimulation on Monocytes-derived dendritic cells (MDCs). MDCs were generated by in vitro culture of CD14^+^ monocytes for 6 days as described in methodology. MDCs were then stimulated with above-mentioned UV inactivated culture supernatants for 48 h and assayed for CD80, CD83 and CD86 expression by flow cytometry assay (lower panel). The percentage of MDCs expressing CD80, CD83 and CD86 and mean fluorescence intensity (MFI) were shown. Data presented are mean of two independent experiments (*n* = 4 donors). Error bars indicate standard error of the mean. *p < 0.05; **p < 0.01; ***p < 0.001 when compared with MDCs stimulated with supernatant from untreated H7N9-infected cells by one-way ANOVA. **c** upper panel indicated the protocol for MDCs stimulation of T-cell proliferation. In vitro derived MDCs were stimulated with above-mentioned supernatants for 48 h. The cells were collected and mixed with CSFE-labelled allogeneic CD3^+^ T cells at 1:3 ratio and co-cultured for 4 or 6 days. Lower panel presents the percentage of proliferated T cells determined by flow cytometry (*n* = 6 donors). Error bars indicate standard error of the mean. *p < 0.05; **p < 0.01 when compared with T cells co-cultured with MDCs stimulated with supernatant from untreated H7N9-infected cells by one-way ANOVA

Activation of cell death pathways by influenza virus may alter the host cell immune responses^38,39^. To elucidate how the concurrent activation of apoptosis and necroptosis pathways regulates immune response of monocytes in H7N9 infection, we first investigated the expression of cytokine and chemokine upon apoptosis or necroptosis modulation. As demonstrated in Fig. 6, pan-caspase inhibition by IDN increased the early responses of *IFNA/B*, proinflammatory cytokine (*IL6* and *TNFA*), and chemokine (*MIP1A/B* and *RANTES*) at 6hpi. IDN treatment also increased the expression of GM-CSF and co-stimulatory molecule CD80 in H7N9-infected monocytes (Fig. 6). In contrast, inhibition of RIPK3 by kinase inhibitor GSKs significantly reduced the expression of *IL1B* and substantially reduced the expression of *IFNA/B, IL6, TNFA, MIP1A/B*, *RANTES*, GM-CSF and CD80 (Fig. 6). This data suggested the early innate immune responses required RIPK3 kinase activity, while caspase activities suppressed H7N9-induced immune response in monocytes. IDN, GSKs, or NSA treatment alone or in combination showed no toxicity to mock-infected monocytes (Supplementary Fig. S2a) and did not impact cytokine expression in mock-infected monocytes except for slight induction of *MIP1A* and *MIP1B* gene by IDN treatment (Supplementary Fig. S2b). Next, we studied how H7N9-infected monocytes may affect the bystander uninfected monocytes and monocyte-derived dendritic cells (MDCs). Culture supernatants from H7N9- and mock-infected monocytes with or without treatment were collected (Supplementary Fig. S3a, b) and added to freshly purified monocytes after UV-inactivation of the virus (Fig. 7a, upper illustration). 72 h later, flow cytometry analysis of the monocytes demonstrated that the culture supernatant from H7N9-infected monocytes with IDN treatment significantly increased the expression of co-stimulatory molecule CD80, CD83 and CD86 (Fig. 7a). Similar experiments on MDCs (Fig. 7b, upper illustration) showed that culture supernatant from H7N9-infected monocytes with IDN treatment similarly augmented the expression of CD80, CD83 and CD86 in MDCs (Fig. 7b). In contrast, GSKs-treatment lowered the expression of CD80, CD83, and CD86 in MDCs (Fig. 7b). As a positive control, LPS (100 ng/ml) induced significantly higher level of CD83 and CD86 in MDCs (Supplementary Fig. S4a). Furthermore, the MDCs stimulated by the above-mentioned culture supernatants for 48 h were co-cultured with freshly isolated, CFSE labeled, allogeneic CD3^+^ human T cells (Fig. 7c, upper illustration). Again, our data suggested that MDCs that previously exposed to supernatant from IDN-treated H7N9-infected monocytes significantly promoted T-cell proliferation (Fig. 7c and Supplementary Fig. S4b−d).

## Discussion

Influenza virus infection and replication induce cell death causing the destruction of airway tissue and induction of inflammatory response^13,40^. The type and consequence of cell death are closely associated with disease development, which are virus strain and cell type dependent^41^. In this study, we demonstrated that avian influenza A H7N9 virus infection of human monocytes concurrently activated apoptosis and necroptosis. A morphology that compatible with apoptotic cell death was readily detected in H7N9-infected monocytes, which could be promoted towards necroptosis in the presence of caspase inhibition. In H7N9-infected monocytes, RIPK3 activation associated with the upregulation of type I interferons and inflammatory cytokine/chemokine, while caspases activation suppressed the expression of these cytokine/chemokines.

Upon H7N9 infection, monocytes deteriorated progressively in terms of viability and cell morphology. TUNEL labeling, activation of caspase-3 and TEM images^42^ indicated activation of apoptosis activation. Caspase-8 and caspase-9 cleavage, upregulation of death receptors and ligands, as well as the increased expression of PUMA and NOXA collectively suggested that both extrinsic and intrinsic apoptosis pathways were activated. This is consistent with previous studies of other subtypes of influenza virus in which viral proteins and inflammatory cytokines induced apoptosis through intrinsic and extrinsic pathways, respectively^34,43–45^. On the other hand, upregulation of RIPK1/RIPK3 expression, interaction between RIPK1/RIPK3/MLKL, and the phosphorylation of MLKL indicated necroptosis activation in parallel with apoptosis. This finding is in line with previous studies that demonstrated influenza H1N1 and H3N2 viruses simultaneously activate apoptosis and necroptosis pathways in infected cells^2,18,20^.

In PR8/H1N1-infected macrophage THP-1 cell line, pMLKL was only detected when the cells were treated with pan-caspase inhibitor^46^. This was in agreement with the notion that activated caspase-8 mediated the degradation of RIPK3^47^. In the current study, we demonstrated that despite the protein levels of RIPK1 and RIPK3 were decreased at 12hpi, phosphorylation of MLKL was evident upon H7N9 infection. This finding implied that the activated caspase-8 activity did not completely suppress RIPK1/3^48,49^ and simultaneous apoptosis and necroptosis were triggered in H7N9-infected monocytes. Interestingly, an apoptotic morphology was more readily detected among the H7N9-infected monocytes under TEM. This could potentially due to the sample preparation procedures, during which the necroptotic cells were lost more easily owing to the nature of necroptosis.

In PR8/H1N1-infected MEFs, RIPK3 kinase activity is only needed for necroptosis, but not apoptosis, while RIPK3 protein is required for interacting with FADD to induce apoptosis^20^. The same mechanism may apply to H7N9-infected monocytes. Treatment with RIPK3-specific kinase inhibitors GSK’872 and GSK’843^50^ completely suppressed MLKL phosphorylation, but the cells still underwent apoptosis suggesting that H7N9-induced apoptosis in monocytes was independent of RIPK3 kinase activity. Whether and how the RIPK3 protein involved in H7N9-induced apoptosis will require further study by genetic ablation of the RIPK3 gene^20,21^. Our data showed that NEC-1, which inhibits RIPK1 kinase activity^51,52^, transiently blocked MLKL phosphorylation at early time points, suggesting that RIPK1 may play a minor role in the activation of necroptosis in H7N9-infected monocytes.

The rapid and severe cell death observed in H7N9-infected monocytes may correlate with the reduction in CD14^+^ monocytes counts and decreased antigen-presenting function reported in H7N9 human infections^53^, which also corroborated with the lymphoid apoptosis and lymphopenia in H5N1 patients^54^. Moreover, previous studies also showed that influenza virus infection caused alveolar macrophages depletion leading to uncontrolled virus replication and development of severe disease in animal models^55–57^. Although monocytes are different from alveolar macrophages, H7N9-induced monocyte cell death could similarly affect the host immune response and the outcome of infection.

In addition to the direct cytopathology, activation of apoptosis and necroptosis may differentially modulate the inflammatory responses to influenza infection^14,41,58^. The expression of *IFNA/B* and proinflammatory cytokine/chemokine was significantly enhanced by IDN treatment but inhibited by RIPK3 inhibitors. These results suggested that the RIPK3 kinase activity promoted the innate immune response to H7N9 infection, which was suppressed by caspase activation. Similarly, inhibition of proinflammatory cytokine responses by early activation of apoptosis in influenza-infected porcine alveolar macrophages was previously reported^4^. The non-immunogenic feature of apoptosis is also realized through the formation of apoptotic bodies, which prevent excessive inflammatory response^14^. In contrast, necroptosis, being immunogenic by releasing DAMPs from the dead cells, promotes inflammation and stimulates immune cell activation. RIPK3 activation has been reported to promote innate immune responses independent of necroptosis in H1N1-infected alveolar macrophages^42^, LPS-induced bone-marrow-derived macrophages^59^, and H7N9-infected mice^60^. In our monocyte model, our results suggested that RIPK3 activation was involved in both necroptosis and activation of immune response.

In terms of both cell death and immune responses, the consequence of influenza virus-activated necroptosis and apoptosis is not conclusive. RIPK3-mediated immune responses were suggested to be either detrimental^60^ or beneficial to influenza virus-infected animals^21^. Divangahi and colleagues^42^ showed that RIPK3 activity is important in promoting type I interferon induction in alveolar macrophage infected with influenza virus in vivo and in vitro. Mice deficient in RIPK3 (*Ripk3*−/−) are highly susceptible to PR8/H1N1 virus infection, exhibiting heightened morbidity and mortality. However, Xu et al.^60^ demonstrated that RIPK3-mediated inflammatory response contributed to disease severity and lethality in H7N9-infected mice. The mechanism was independent of necroptosis, but rather through caspase-1/IL-1β signaling-mediated overproduction of cytokine/chemokine and heightened inflammatory infiltration in lung tissue. By genetic knockout of RIPK3, they showed a significantly reduced level of proinflammatory responses in the lung of *Ripk3*−/− mice along with higher survival and less weight loss. This report is in agreement with our previous observations in H7N9-infected wild type BALB/c mice which showed high levels cytokine production and severe lung inflammatory damage, and consequently, a reduced adaptive antibody responses with high mortality^26,31^. The detrimental effect of RIPK3 activation due to cell death has also been demonstrated in mice deficient in cIAP2 gene^18^, in which RIPK1/RIPK3-mediated necroptosis in airway epithelial cells was associated with an increased lethality. Moreover, the inconsistent outcome of ZBP1/DAI deficiency on the survival of PR8/H1N1-infected mice was also reported^2,12^. Comparing with 2009 pandemic H1N1 virus which is less virulent than H7N9, we previously showed that H7N9 virus infected human monocytes much more effectively than that of A(H1N1)pdm09 virus and, and elicited significantly lower expression of monocyte differentiation markers^31^. In this current study, our result suggests that H7N9-induced inflammatory responses and RIPK3 activation favor the immune activation of antigen-presenting cells, while caspase-mediated cell death does not.

In conclusion, our study demonstrated that H7N9 rapidly activated apoptosis and necroptosis pathways in human monocytes, in which RIPK3 activated immune responses, which was restrained by caspase activation. Prolonged monocyte survival by inhibition of both apoptosis and necroptosis maybe beneficial to host immune responses. Further studies will be needed to investigate the approaches to manipulate the apoptosis and necroptosis pathways to improve the outcome of H7N9 infection in vivo.

## Disclaimer

The sponsors had no role in the design and conduct of the study, in the collection, analysis and interpretation of data, or in the preparation, review or approval of the manuscript.

## Supplementary information

Supplementary Figure S1.

Supplementary Figure S2.

Supplementary Figure S3.

Supplementary Figure S4.
