# Supplementary material for: H7N9 influenza A virus activation of necroptosis in human monocytes links innate and adaptive immune responses
Source: Cell Death Dis. 2019 Jun 5;10(6):442. doi: 10.1038/s41419-019-1684-0 (PMC6549191; doi:10.1038/s41419-019-1684-0)
Supplement: Supplementary file 1 — Supplementary Figure S1. [file 41419_2019_1684_MOESM1_ESM.pdf]

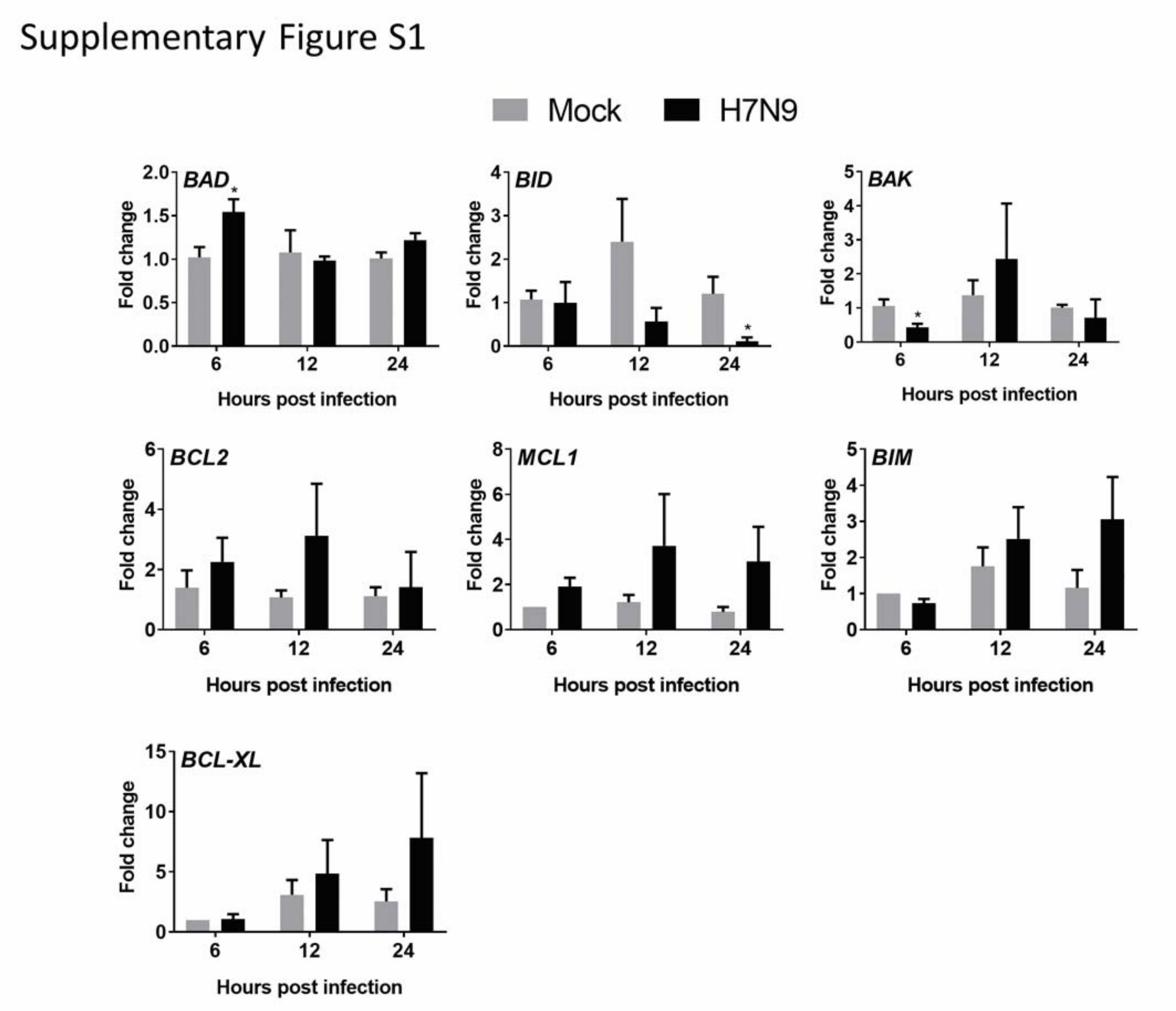

Supplementary Fig. S1 Real time RT-PCR determined relative expression of apoptosis regulating genes: *BAD, BID, BAK, BCL-2, MCL1, BIM* and *BCL-XL* in H7N9-infected monocytes at 6, 12 and 24hpi. Fold of changes compared to mock-infected monocytes at 6hpi which was taken as 1. Data represented mean of two independent experiments (n = 4 donors). Error bars indicate standard error of the mean. * $p< 0.05$ when compared with mock-infected cells by student's t-test.
