# Supplementary material for: H7N9 influenza A virus activation of necroptosis in human monocytes links innate and adaptive immune responses
Source: Cell Death Dis. 2019 Jun 5;10(6):442. doi: 10.1038/s41419-019-1684-0 (PMC6549191; doi:10.1038/s41419-019-1684-0)
Supplement: Supplementary file 2 — Supplementary Figure S2. [file 41419_2019_1684_MOESM2_ESM.pdf]

Supplementary Figure S2

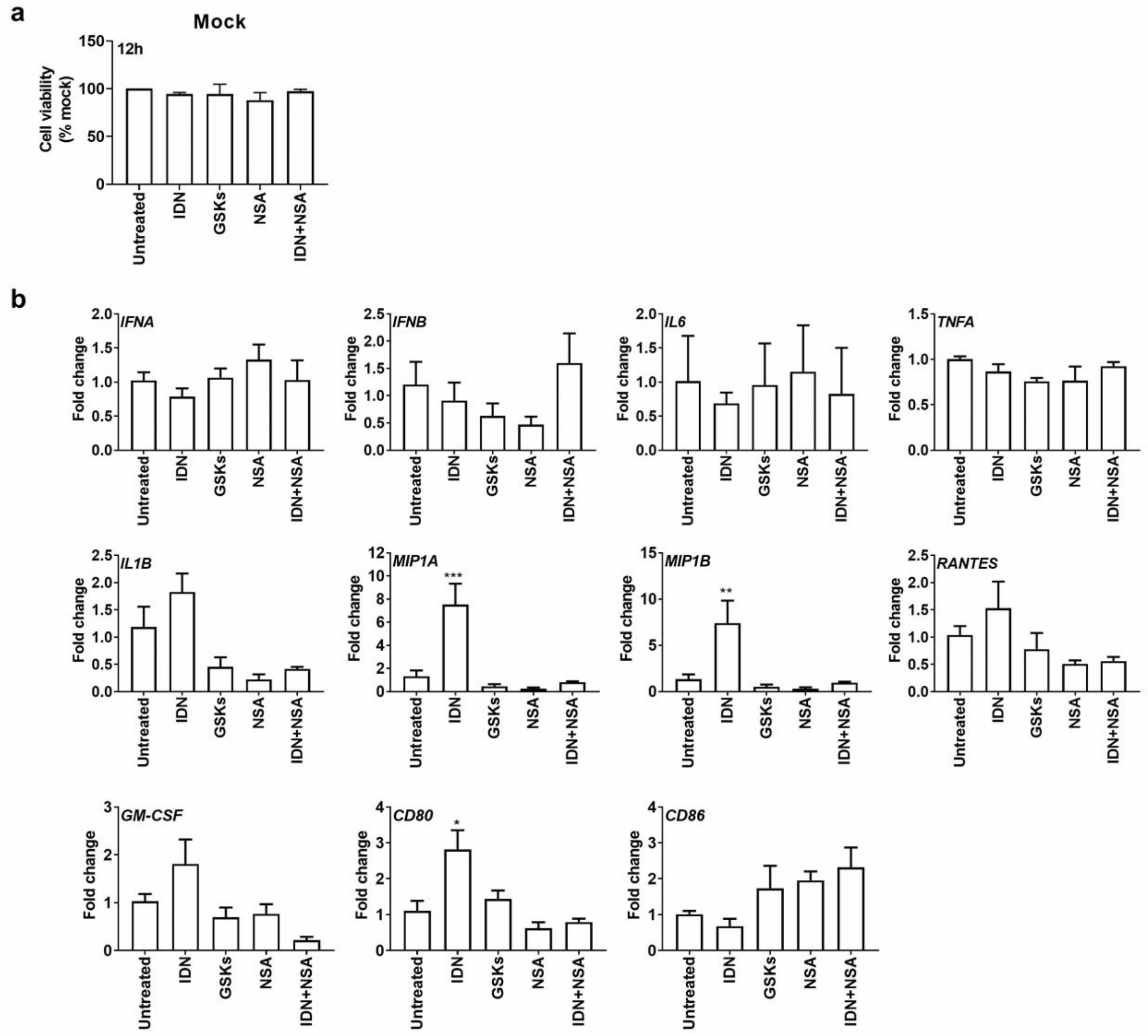

Supplementary Fig S2 a Cell viability of mock-infected monocytes treated with different inhibitors for 12 hours determined by CellTiter-Glo assay. **b** Relative gene expression determined by real time RT-PCR in mock-infected monocytes after 6 hours treatment with different inhibitors. Fold of changes compared with untreated control. Data represented mean of two independent experiments (n = 4 donors).
