# Supplementary material for: H7N9 influenza A virus activation of necroptosis in human monocytes links innate and adaptive immune responses
Source: Cell Death Dis. 2019 Jun 5;10(6):442. doi: 10.1038/s41419-019-1684-0 (PMC6549191; doi:10.1038/s41419-019-1684-0)
Supplement: Supplementary file 3 — Supplementary Figure S3. [file 41419_2019_1684_MOESM3_ESM.pdf]

Supplementary Figure S3

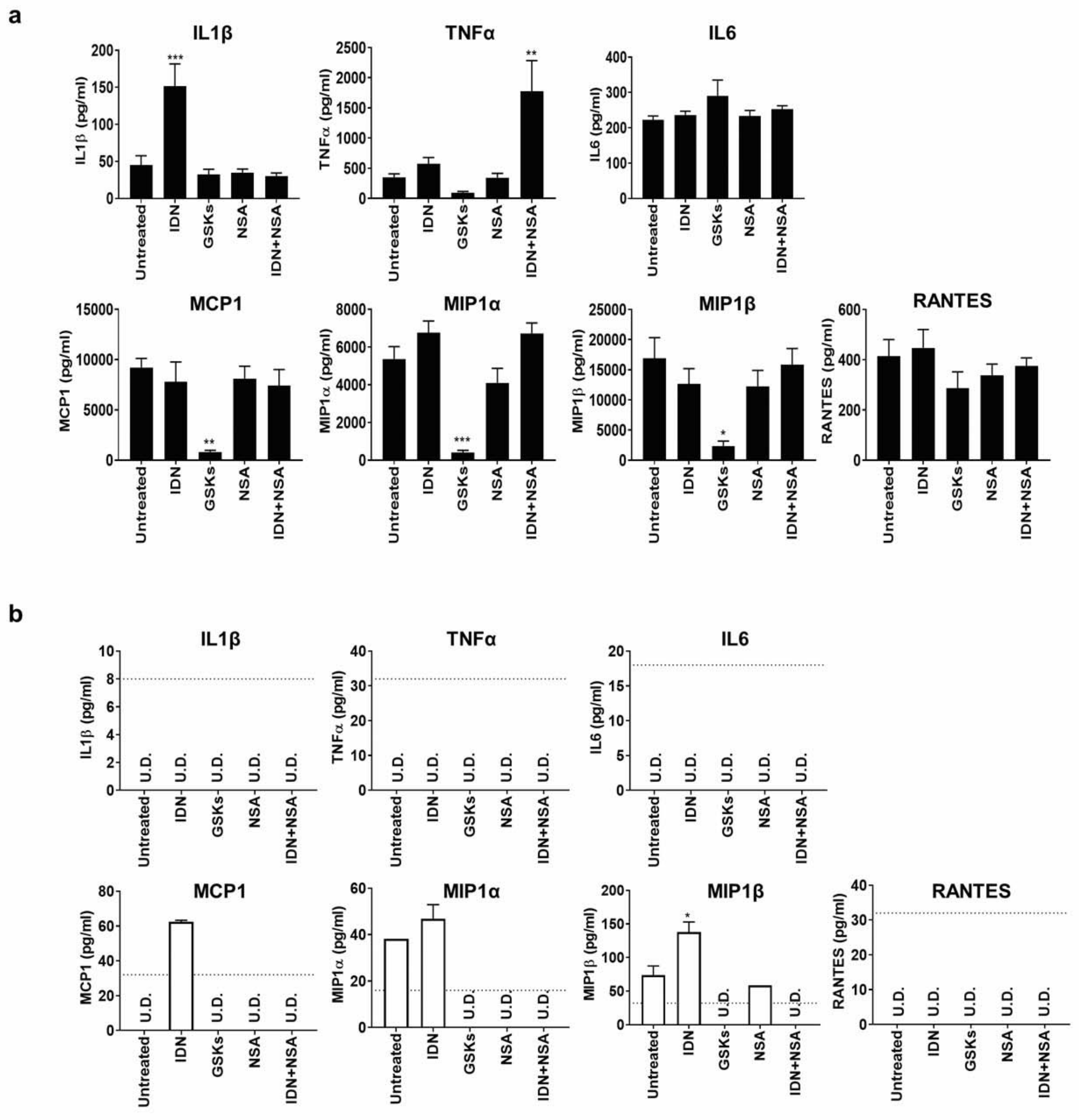

Supplementary Fig. S3 Cytokine/chemokine concentration in the culture supernatant at 24hpi determined by ELISA assay. **a** H7N9-infected and **b** mock-infected monocytes culture supernatant were collected at 24hpi and tested using ELISA kit (R&D system) (n = 4-6 donors). U.D. means undetectable. Dashed lines indicate the detection limit of the assay kits. Error bars indicate standard error of the mean. * $p< 0.05$; ** $p< 0.01$; *** $p< 0.001$ when compared with untreated culture supernatant by one-way ANOVA.
