# Supplementary material for: H7N9 influenza A virus activation of necroptosis in human monocytes links innate and adaptive immune responses
Source: Cell Death Dis. 2019 Jun 5;10(6):442. doi: 10.1038/s41419-019-1684-0 (PMC6549191; doi:10.1038/s41419-019-1684-0)
Supplement: Supplementary file 4 — Supplementary Figure S4. [file 41419_2019_1684_MOESM4_ESM.pdf]

Supplementary Figure S4

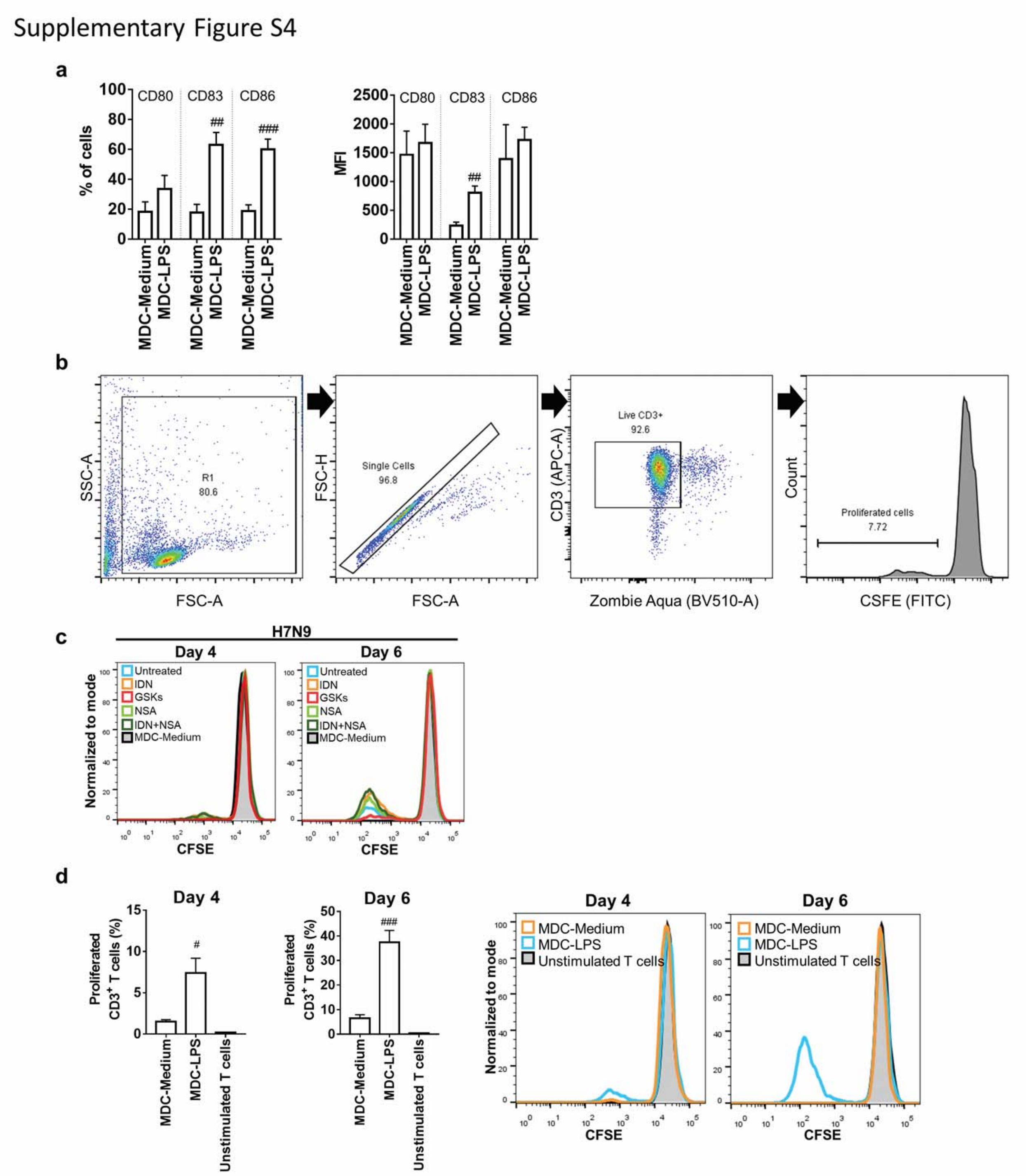

Supplementary Fig. S4 a Flow cytometry determined percentage of cells expressing CD80, CD83, CD86 and MFI of MDCs after monocytes stimulated by LPS (100ng/ml) for 48 hours (n = 4 donors). ## *p*< 0.01; ### p<0.001 when compared with MDCs stimulated with plain medium by one-way ANOVA. **b** Flow cytometry gating strategy for T cell proliferation analysis. **c** Representative flow

cytometry histogram of T cell proliferation induced by MDCs stimulated by culture supernatant from H7N9-infected monocytes. **d** Percentage of LPS matured MDCs induced T cell proliferation. MDCs stimulated with LPS or MDCs medium only for 48 hours. The cells were collected and mixed with CSFE-labelled $CD3^+$ T cells at 1:3 ratio and co-cultured for 4 or 6 days. T cell proliferation were determined by flow cytometry. Data presented were the percentage of proliferated T cells determined by flow cytometry, and representative flow histogram (n = 6 donors). # $p< 0.05$; ### $p<0.001$ when compared with T cells co-cultured with MDCs stimulated with plain medium by one-way ANOVA.
